# Which stem cells will eventually translate to the clinics for treatment of diabetes?

**DOI:** 10.1186/s13287-020-01718-3

**Published:** 2020-06-03

**Authors:** Deepa Bhartiya, Subhan Ali Mohammad

**Affiliations:** Stem Cell Biology Department, ICMR-National Institute for Research in Reproductive Health, Jehangir Merwanji Street, Parel, Mumbai, 400012 India

**Keywords:** Pancreas, Stem cells, Diabetes, Regeneration, VSELs, MSCs, hES cells, iPS cells

## Abstract

Human embryonic stem (hES) cells have been around for more than two decades now. It was expected that hES/iPS (induced pluripotent stem) cells will quickly translate to the clinics to treat diabetic patients and to obtain gametes in vitro for infertile couples. However, there is no breakthrough yet in either of the fields although considerable progress has been made. Research efforts are ongoing to obtain an insight into the gene expression changes associated with directed differentiation of hES/iPS cells. Autologous bone marrow/cord blood mononuclear cells’ therapy has also failed to show any regenerative potential and only remains as a standard method of care for blood diseases. Only mesenchymal stem cells (MSCs) have shown promise in the clinics to alleviate diabetic symptoms. But MSCs are stromal cells with no regenerative properties; rather “paracrine providers”, pericytes/stromal cells, better known for their trophic, immuno-modulatory, and anti-inflammatory properties and thus best termed as mesenchymal stromal cells (MSCs). Autologus bone marrow cells enriched for hematopoietic stem cells have no potential to cross boundaries and transdifferentiate into other lineages including endodermal pancreatic cells. Endogenous, pluripotent, very small embryonic-like stem cells (VSELs) emerge as the most likely endogenous stem cell candidates to regenerate adult diabetic pancreas. Transplanted MSCs provide a healthy paracrine support required for endogenous/ resident VSELs to differentiate into acinar cells and islets in a diabetic pancreas to enable restoration of homeostasis. Our recently published study shows that VSELs exist and can be enriched from intact mouse pancreas as well as from the islets and increase in numbers in diabetic pancreas. Providing “regenerative pressure” by subjecting diabetic mice to partial pancreatectomy stimulated the VSELs to undergo differentiation into various cell types in an attempt to restore homeostasis. Double-blinded, placebo controlled clinical trials need to be undertaken to evaluate the efficacy of transplanting MSCs in diabetic patients with conviction since now underlying fine play of endogenous VSELs and niche providing MSCs has emerged.

## Main text

Almost 9–10% of world population suffers from type I and type 2 diabetes that results in approximately 4 million deaths annually [1]. Transplanting beta islets holds lot of promise but is associated with life-long use of immuno-suppressants, and availability of islets for therapy remains a bottleneck [2]. On the other hand, use of stem cells can provide a renewable source of cells for therapy. It is now more than two decades since human embryonic stem (hES) cell lines were successfully derived in vitro for the first time by Thomson’s group from USA [3], and it was speculated that cure for diabetes will be one of the first applications. Later, Yamanaka’s group from Japan identified the factors to reprogram somatic cells into induced pluripotent stem (iPS) cells [4]. Hopes soared high because iPS cells besides having lesser associated ethical concerns also took care of immunological issues. The report of Key Opinion Leaders, working in the field of stem cells to replace beta cells, published in 2018 on the progress made over the years towards stem cell-derived beta cells concluded that much more work still remains to be done before moving to the clinics [5]. With the advent of gene-editing technologies, it is hoped to obtain ultimate personalized medicine beta cell product from iPS cells [6].

Melton’s group recently published an elegant study in Nature [7] where they have described the dynamics of gene expression changes by high resolution sequencing approach when pluripotent stem cells differentiate into beta cells in vitro. It is being realized that transplantation of pancreatic progenitors obtained from hES/iPS cells does not yield beta cells efficiently and the underlying molecular control of the differentiation process is not well understood [7]. This is the basic underlying reason why the clinical trial initiated in 2014 by ViaCyte, a San Diego-based company, has not yet yielded a breakthrough although significant progress has been made in the field (https://www.cellandgene.com/doc/who-will-win-the-regenerative-medicine-for-diabetes-race-0001, https://ipscell.com/2019/09/viacyte-qa-crispr-tx-quick-progress-ongoing-diabetes-trial-more/). It was hoped that pancreatic progenitors obtained by the differentiation of hES/iPS cells will differentiate into beta cells efficiently when transplanted in the patients, but this did not materialize. Recently, Viacyte has collaborated with CRISPER Therapeutics to prepare gene-edited cells that will not be attacked by the host immune system (https://ipscell.com/2019/09/viacyte-qa-crispr-tx-quick-progress-ongoing-diabetes-trial-more/).

We had recently discussed current status of clinical translation of various types of stem cells for regenerative medicine [8]. Use of adult, autologous stem cells from bone marrow/cord blood (hematopoietic stem cells) has now been limited to treating only blood and immune system diseases. Besides treating diabetes, differentiating gametes from the stem cells was also considered an easily achievable target for infertile couples. But with no success yet in both the fields however, enthusiasm persists now to carry out gene editing of the pancreatic progenitors obtained by differentiation of hES/iPS cells for cell therapy (https://ipscell.com/2019/09/viacyte-qa-crispr-tx-quick-progress-ongoing-diabetes-trial-more/).

Our group has remained focused on endogenous stem cells present in adult tissues including pancreas for regenerative medicine [8, 9]. These include pluripotent very small embryonic-like stem cells (VSELs) and lineage restricted, tissue-committed stem cells (TCSCs) also termed “progenitors” which include, e.g., hematopoietic stem cells (HSCs) in the bone marrow/cord blood, spermatogonial stem cells (SSCs) in the testes, ovarian stem cells (OSCs) in the ovary, and so on. VSELs self-renew and give rise to HSCs/SSCs/OSCs by undergoing asymmetrical cell divisions whereas the TCSCs (progenitors) further differentiate into tissue-specific cell types [10]. HSCs differentiate into various types of blood cells, SSCs differentiate into sperm, and OSCs differentiate into oocytes. The presence of VSELs in the cord blood/bone marrow has now been confirmed by 20 independent groups across the world [11]. Only VSELs possess “true” regenerative potential, can differentiate into multiple cell types, and cross boundaries whereas TCSCs (progenitors) are tissue committed and do not have the ability to transdifferentiate. Pancreas harbors pluripotent VSELs along with slightly bigger TCSCs termed pancreatic stem cells (PSCs) which are multipotent and express PDX-1 and cytoplasmic OCT-4 [12, 13]. To conclude, VSELs are the most primitive, pluripotent stem cells present in multiple adult tissues and serve as a back-up pool for the TCSCs. A delicate interplay between these stem cells and their niche “microenvironment” results in life-long tissue homeostasis.

A role of VSELs/PSCs in regeneration of normal mouse pancreas after partial pancreatectomy was delineated earlier [12]. We recently published our findings on VSELs/PSCs in a diabetic, streptozotocin-treated mouse model [13]. An innovative protocol was developed to enrich the stem cells from the pancreas wherein after enzymatic digestion the majority of cells were pelleted down by spinning at 1000 rpm. At this speed, the stem cells remained buoyant and were later enriched from the supernatant by spinning at 3000 rpm. This simple and robust approach can help enrich VSELs from any solid organ [14]. Then using flow cytometry, 2–6μm, viable (7AAD neg) VSELs with a surface phenotype of LIN-CD45-SCA-1+ were studied and a 10-fold enrichment of VSELs was observed in the 3000 rpm pellet. Using similar protocol, we also detected and characterized VSELs from a pure population of pancreatic islets. Diabetic mice had increased numbers of VSELs in the pancreas compared to normal control mice (% events representing VSELs per 5 lakhs analyzed increased from 0.725 to 1.142% by flow cytometry, 15). This increase in VSEL numbers in the diabetic pancreas was similar to their increase in the testis and ovary post chemotherapy [15]. VSELs increase in numbers in response to any kind of stress in an attempt to restore homeostasis including the damage inflicted by STZ or busulphan. Thus, rather than hES/iPS cells grown in a Petri dish, it is the endogenous pluripotent VSELs that have the true potential to regenerate adult diabetic pancreas. We published evidence to show that when a diabetic pancreas was subjected to partial pancreatectomy, endogenous VSELs differentiated into acinar cells and later into various cell types that make up an islet resulting in their neogenesis [13]. It is likely that diabetes induced by STZ treatment instead of affecting the stem cells possibly affects the niche providing cells to the VSELs [13]. This is based on the observation that VSELs remain functional and increase in numbers in the STZ-treated diabetic pancreas but are unable to differentiate since possibly the niche gets compromised by STZ. We have earlier reported that VSELs are not affected by busulphan treatment, rather increase in numbers, but the Sertoli cells (which provide a “niche” to the testicular stem cells) get affected based on the results of a microarray study [15, 16]. Transplanting healthy niche cells (Sertoli or mesenchymal cells) could completely restore spermatogenesis from the endogenous VSELs [16] whereas transplanting induced pluripotent stem cells (iPS) cells by another group failed to restore spermatogenesis [17] in chemoablated mouse testes. Thus, it is crucial to think of stem cells and their niche microenvironment together while aiming to achieve regeneration. Scientific community is unsuccessfully transplanting tissue-specific progenitors obtained by differentiation of hES/iPS cells with an aim to achieve regeneration, but the defect lies in the niche. A compromised niche will never support effective maturation of transplanted progenitors!

Mesenchymal stem/stromal cells (MSCs) have been used for clinical trials [18] and in rodent models [19] where they ameliorate the diabetic symptoms. Currently, only MSCs have reached the clinics and show the potential to regenerate diabetic pancreas; however, the underlying mechanism for the beneficial effects is still not clear. It needs to be understood that MSCs do not differentiate into pancreatic cell types; rather, they provide the required paracrine support to the endogenous VSELs to differentiate and restore homeostasis of the diseased organ. To conclude, the only way forward to regenerate diseased pancreas is by transplanting MSCs which do not regenerate but help restore pancreatic function by being a “paracrine provider” and thus supporting endogenous VSELs/PSCs differentiation and thereby regeneration.

But a huge disbelief exists in the field, and the very presence of stem cells is denied in the adult pancreas [20]. VSELs struggle to get wider recognition [21]. It is hoped our robust protocol to enrich pluripotent stem cells from adult tissues [13, 14] will be acknowledged by the scientific community. Pluripotent VSELs being present in multiple adult tissues have the ability to regenerate them compared to hES cells derived from inner cell mass of blastocyst stage embryos or iPS cells obtained by reprogramming somatic cells. Both hES and iPS cells tend to differentiate into their fetal counterparts. As a result of this shortfall to tap the regenerative potential of iPS cells, investigators in Japan are now planning to use iPS cells as a source of growth factors and cytokines and also contemplating their allogeneic rather than autologous use. Several groups have reported differentiation of iPS cells into MSCs in vitro [22, 23]. But MSCs can be easily obtained from several sources and have the potential to provide paracrine support and ameliorate disease symptoms. There is no need to reprogram somatic cells into iPS cells for differentiation into MSCs for transplantation [24]. Recently, it was reported by Scholer’s group that OCT-4 is not crucial for reprogramming somatic cells to pluripotent state [25]. Then what are iPSCs? It was always believed and proposed by the same group that OCT-4 is the most crucial gene and sufficient to induce pluripotency in somatic cells [26]! It has been earlier suggested that a small sub-population of pluripotent stem cells expressing Oct-4, Sox-2, and Nanog (MUSE cells) among the human skin fibroblast start growing as iPSCs, thereby questioning reprogramming of somatic cells to pluripotent state [27]. It is likely that a sub-population of pluripotent stem cells (MUSE cells or VSELs) start expanding in culture during iPSC derivation rather than reprogramming of fully committed somatic cells. These pluripotent stem cells are highly scarce in nature, and this possibly explains the inefficient derivation of iPS cell colonies while reprogramming somatic cells. Definitely, there is a lot more to learn in the field of stem cells.

To conclude, VSELs exist in the adult pancreas and also in the enriched fraction of pancreatic islets. They increase in numbers but are unable to differentiate and restore homeostasis in the diabetic pancreas [12]. The root cause of diabetes seems to be a functionally compromised niche to the endogenous VSELs. Effective therapy to treat diabetes (regenerate diabetic pancreas) will involve transplanting healthy, niche providing mesenchymal stromal cells. This will enable endogenous VSELs to function normally (differentiate into islets) and help regenerate diabetic pancreas and restore tissue homeostasis.

## Data Availability

Not applicable.

## Abbreviations

VSELs: Very small embryonic-like stem cells
PSCs: Pancreatic stem cells
HSCs: Hematopoietic stem cells
MSCs: Mesenchymal stem/ stromal cells
hES cells: Human embryonic stem cells
iPS cells: Induced pluripotent stem cells
STZ: Streptozotocin
